# Galanin Receptors (GALR1, GALR2, and GALR3) Immunoexpression in Enteric Plexuses of Colorectal Cancer Patients: Correlation with the Clinico-Pathological Parameters

**DOI:** 10.3390/biom12121769

**Published:** 2022-11-27

**Authors:** Jacek Kiezun, Marta Kiezun, Bartlomiej Emil Krazinski, Lukasz Paukszto, Anna Koprowicz-Wielguszewska, Zbigniew Kmiec, Janusz Godlewski

**Affiliations:** 1 Department of Human Histology and Embryology, School of Medicine, University of Warmia and Mazury in Olsztyn, Warszawska Street 30, 10-082 Olsztyn, Poland; 2 Department of Animal Anatomy and Physiology, Faculty of Biology and Biotechnology, University of Warmia and Mazury in Olsztyn, Oczapowskiego Street 1a, 10-719 Olsztyn, Poland; 3 Department of Botany and Nature Protection, Faculty of Biology and Biotechnology, University of Warmia and Mazury in Olsztyn, Plac Łódzki 1, 10-719 Olsztyn, Poland; 4 Department of Histology, Faculty of Medicine, Medical University of Gdansk, Sklodowskiej-Curie Street 3a, 80-211 Gdansk, Poland

**Keywords:** colorectal cancer, galanin receptors, enteric plexuses, immunohistochemistry, prognosis

## Abstract

Galanin (GAL) is an important neurotransmitter released by the enteric nervous system (ENS) neurons located in the muscularis externa and submucosa enteric plexuses that acts by binding to GAL receptors 1, 2 and 3 (GALR1, 2 and 3). In our previous studies, the GAL immunoexpression was compared in colorectal cancer (CRC) tissue and the adjacent parts of the large intestine wall including myenteric and submucosal plexuses. Recently we have also found that expression levels of GALR1 and GALR3 proteins are elevated in CRC tissue as compared with their expression in epithelial cells of unchanged mucosa. Moreover, higher GALR3 immunoreactivity in CRC cells correlated with better prognosis of CRC patients. To understand the distribution of GALRs in enteric plexuses distal and close to CRC invasion, in the present study we decided to evaluate GALRs expression within the myenteric and submucosal plexuses located proximally and distally to the cancer invasion and correlated the GALRs expression levels with the clinico-pathological data of CRC patients. The immunohistochemical and immunofluorescent methods showed only slightly decreased immunoexpression of GALR1 and GALR3 in myenteric plexuses close to cancer but did not reveal any correlation in the immunoexpression of all three GAL receptors in myenteric plexuses and tumour progression. No significant changes were found between the expression levels of GALRs in submucosal plexuses distal and close to the tumour. However, elevated GALR1 expression in submucosal plexuses in vicinity of CRC correlated with poor prognosis, higher tumour grading and shorter overall survival. When myenteric plexuses undergo morphological and functional alterations characteristic for atrophy, GALRs maintain or only slightly decrease their expression status. In contrast, the correlation between high expression of GALR1 in the submucosal plexuses and overall survival of CRC patients suggest that GAL and GALRs can act as a components of local neuro-paracrine pro-proliferative pathways accelerating the invasion and metastasis of cancer cell. The obtained results suggest an important role of GALR1 in submucosal plexuses function during the progression of CRC and imply that GALR1 expression in submucosal plexuses of ENS could be an important predictive factor for CRC progression.

## 1. Introduction

Galanin (GAL) is a principal signalling neuropeptide of the galanin family molecules distributed throughout the central and peripheral nervous system [1,2,3]. GAL is secreted in the enteric nervous system (ENS) as one of the important neurotransmitters secreted by gastrointestinal (GI) plexuses, which participates in its neurons development and protection [4,5]. The main functions of that 30-amino acid molecule in the GI tract are to regulate GI epithelial cells secretion and GI motility directly by acting on muscularis externa smooth muscle cells or indirectly by influencing the secretion of various contracting agents widely distributed in GI tract [6,7,8]. In neurons, GAL exerts a neuroprotective action [9] and it has been suggested that this neuropeptide plays an emerging role in inflammatory bowel diseases [10] and cancerogenesis [11]. In human glioma and pituitary adenoma, GAL acts on tumour-infiltrating immune cells and regulates the tumour microenvironment [12,13]. In previous studies, the GAL protein expression was determined in serum and tissue of colorectal cancer (CRC) patients [12,14,15,16] as well as the parts of the colon wall including myenteric and submucosal plexuses [16].

GAL signalling is mediated via galanin receptors 1, 2, and 3 (GALR1, GALR2, and GALR3), which are members of the G-protein-coupled receptor family [17]. GALR1 and GALR3 transmit GAL signalling by the stimulation of adenylate cyclase whereas the biological activity of GALR2 is linked to phospholipase C [17]. The expression of GALR1 (but not that of GALR2 or GALR3) was shown to be controlled by cAMP via CREB [18]. GALRs trigger both of these signalling pathways to affect many physiological and pathological processes localized in the GI tract such as water balance, nociception, energy balance, neurons regeneration [4,11,19,20], changes in inflammatory cytokine levels [21], GI cancers [1,22]. Our previous study showed that the expression of GALRs proteins was higher in CRC tissue, than in unchanged mucosa cells distantly located from the tumour (GALR2 was not significant, but the tendency was noticed) [1]. Moreover, CRC patients with longer survival and better prognosis exerted a higher immunoreactivity of GALR3 in CRC cells compared to lower expression in unchanged epithelial cells. The distribution of GALR3 was associated with tumour size—T, invasion of regional lymph nodes—N, and distant metastases—M (TNM staging system), as well as with the overall survival of CRC patients [1]. These data suggest that GALR3 can be a biomarker of a CRC patient’s prognosis. However, the expression of GALRs in plexuses of muscularis externa and submucosa close and distantly located to the tumour is still unknown. Therefore, the aim of this study was to evaluate the expression of GALRs in myenteric and submucosal ENS plexuses located proximally and distantly from the CRC tissue and to investigate associations between GALRs immunoexpression in plexuses and clinico-pathological characteristics of patients with CRC.

## 2. Results

### 2.1. Immunoexpression of GALR1, 2 and 3 in Myenteric Plexuses Distantly Located from Cancer Tissue Is not Changed Compared to Immunoreactivity in Myenteric Plexuses in the Vicinity of Cancer Invasion

**Figure 1:**
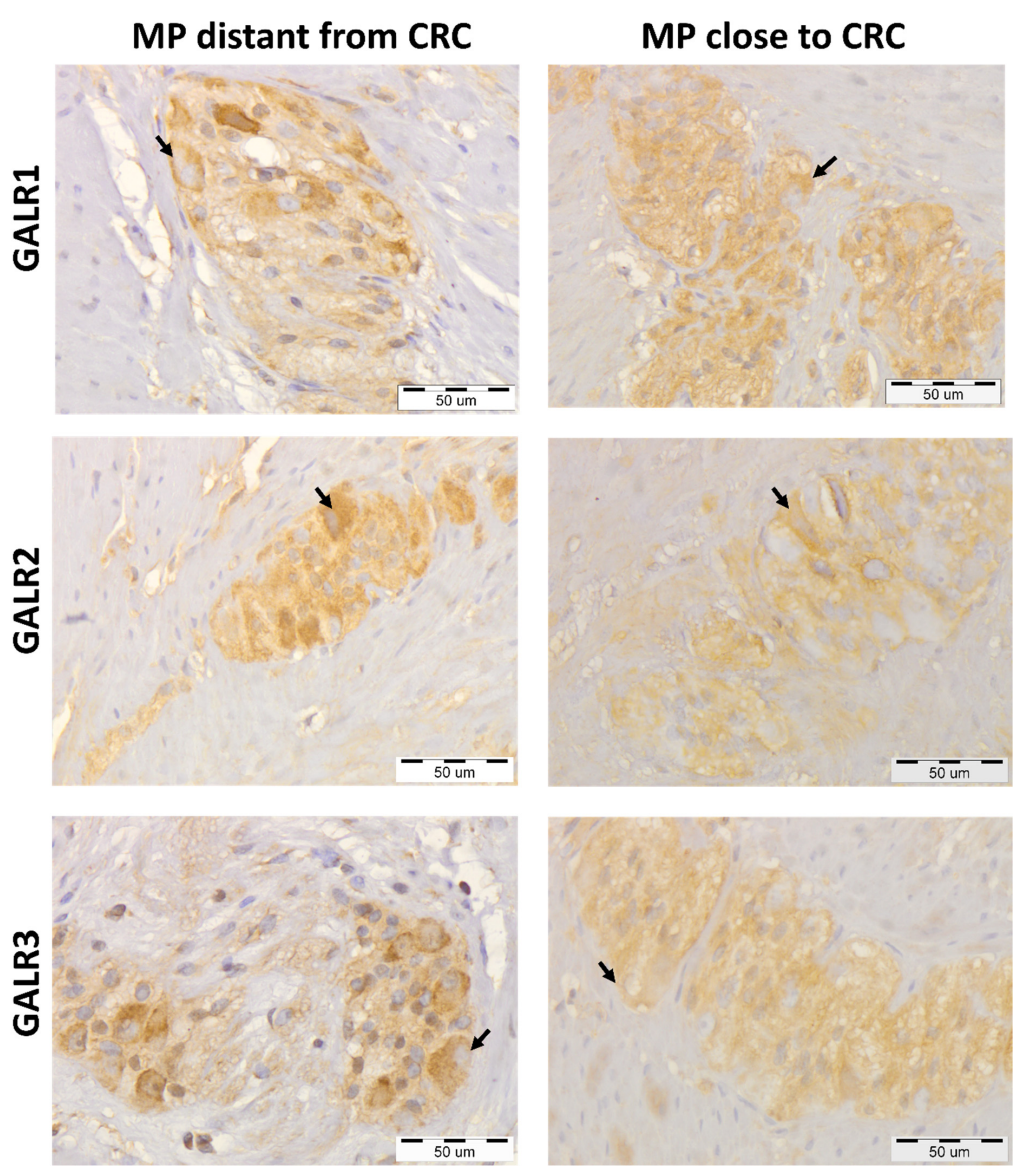
Immunohistochemical expression of galanin receptors (GALR1, GALR2, and GALR3) in myenteric plexuses (MP) distantly located from the colorectal cancer (CRC) tissue compared with their expression in the myenteric plexuses in the vicinity of cancer invasion of the same, representative CRC patients (n = 31). Neurons expressing respective GALRs are marked by arrows. Total magnification: 400× *g*.

The immunoreactivities of GALRs were found in the cell membrane and cytoplasm of the cells of muscularis externa: myenteric plexuses’ cells, smooth muscle cells, and endothelial cells (Figure 1).

**Figure 2:**
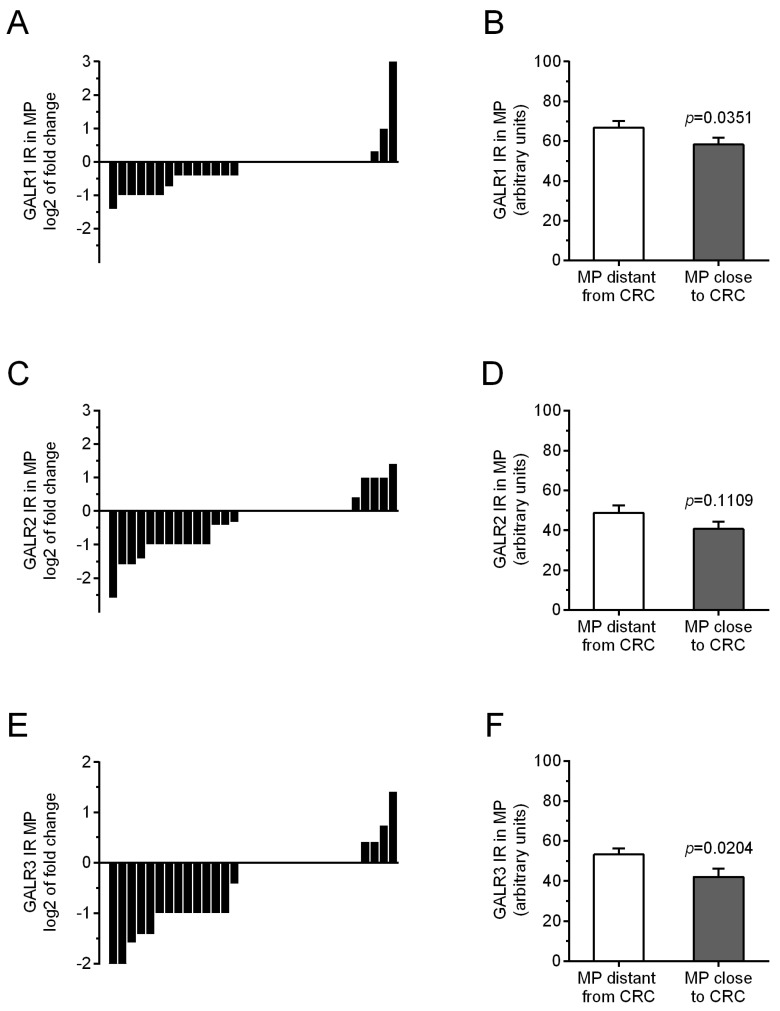
Immunoreactivity (IR) of galanin receptors (GALR1—(**A**), GALR2—(**C**), and GALR3—(**E**)) in myenteric plexuses (MP) distantly and proximity located to cancer invasion in individual colorectal (CRC) patients. The average immunoreactivities of GALRs (GALR1—(**B**), GALR2—(**D**), and GALR3—(**F**)) in myenteric plexuses (MP) located distantly from CRCcells were compared with those in plexuses in the vicinity of cancer cells of colorectal (CRC) patients (n = 31).

Immunoreactivity of all three studied GALRs were similar in myenteric plexuses distantly located from the tumour tissue in comparison with myenteric plexuses located in vicinity to CRC invasion (Figure 2). The average immunoreactivity of GALR1 and GALR3 in muscularis externa plexuses close to the CRC tissue was slightly reduced as compared to the plexuses distant from the tumour, while GALR2 immunoreactivity reminded unchanged (*p* = 0.0351, *p* = 0.0204 and *p* = 0.1109, respectively, Figure 2).

### 2.2. Fluorescent Immunolocalization of GALR1, 2 and 3 in Myenteric Plexuses Distantly and Closely Located to CRC Tissue

**Figure 3:**
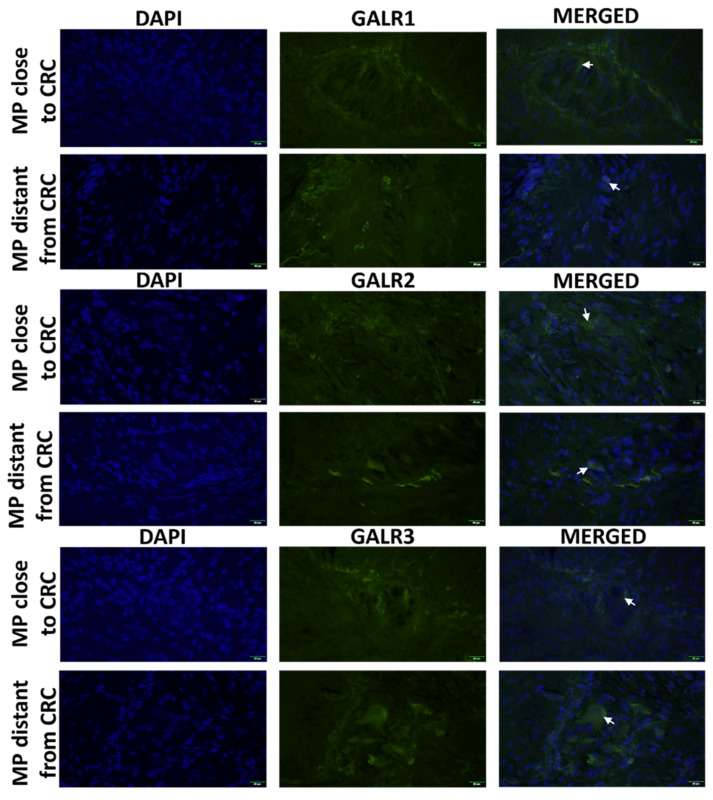
Immunolocalization of galanin receptors (GALR1, GALR2, and GALR3) in myenteric plexuses (MP) close and distant to tumour tissue of CRCpatients (n = 5). Neurons expressing respective GALRs are marked by arrows. Total magnification: 400× *g*.

The immunoexpression of GALR1, 2 and 3 was confined to the cell membrane and cytoplasm of myenteric plexus cells and smooth muscle cells (Figure 3).

### 2.3. Immunoexpression of GALRs in Myenteric Plexuses Is not Associated with Prognosis and Clinico-Pathological Characteristic of CRC Patients

**Figure 4:**
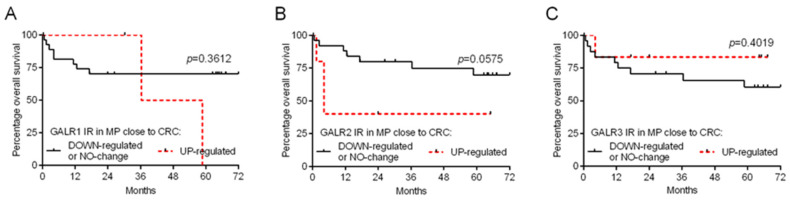
Kaplan–Meier diagrams that show the relative immunoexpression of galanin receptors (GALR1—(**A**), GALR2—(**B**), and GALR3—(**C**)) in myenteric plexuses (MP) regarding the overall survival of colorectal cancer (CRC) patients (n = 31).

We divided CRC patients based on the relative immunoreactivity of GALR specimens into two groups regarded as ‘down-regulated or no change’ (relative GALR immunoreactivity ≤ 1) and ‘up-regulated’ (relative GALR immunoreactivity > 1) to obtain survival curves according to the Kaplan–Meier method. We found that GALR1, GALR2 and GALR3 relative immunoreactivity (comparing myenteric plexuses close to CRC cells vs. plexuses distant from cancer invasion) did not correlate with the overall survival of CRC patients (Figure 4A, B, and C, respectively; Table 1). Similarly, the relative GALRs distribution in myenteric plexuses did not correlate with the demographic and clinico-pathological characteristics of CRC patients (Table 2).

### 2.4. Immunoexpression of GALR1, GALR2 and GALR3 in Submucosal Plexuses of the Large Intestine Wall Located Distantly and Closely to Cancer Tissue

**Figure 5:**
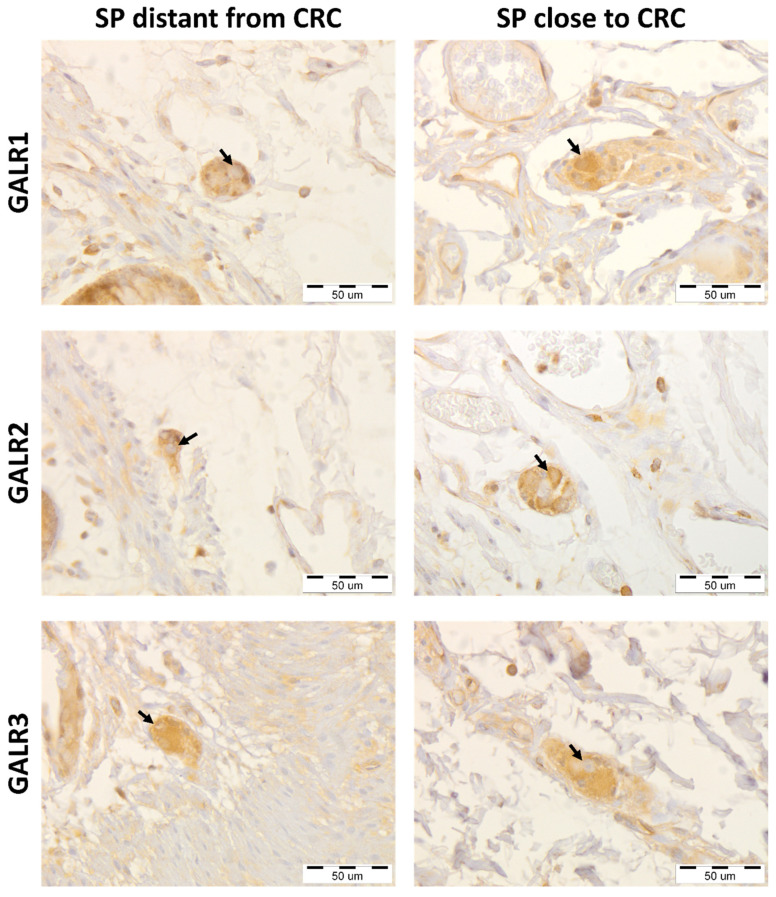
Immunohistochemical expression of galanin receptors (GALR1, GALR2, and GALR3) in submucosal plexuses (SP) distantly located from the colorectal cancer (CRC) tissue compared with their expression in the submucosal plexuses in the vicinity of cancer invasion of the same, representative CRC patients (n = 32). Neurons expressing respective GALRs are marked by arrows. Total magnification: 400× *g*.

The immunoreactivities of GALRs were found in the cell membrane and cytoplasm of the cells present in submucosal plexuses, and stromal cells (Figure 5).

**Figure 6:**
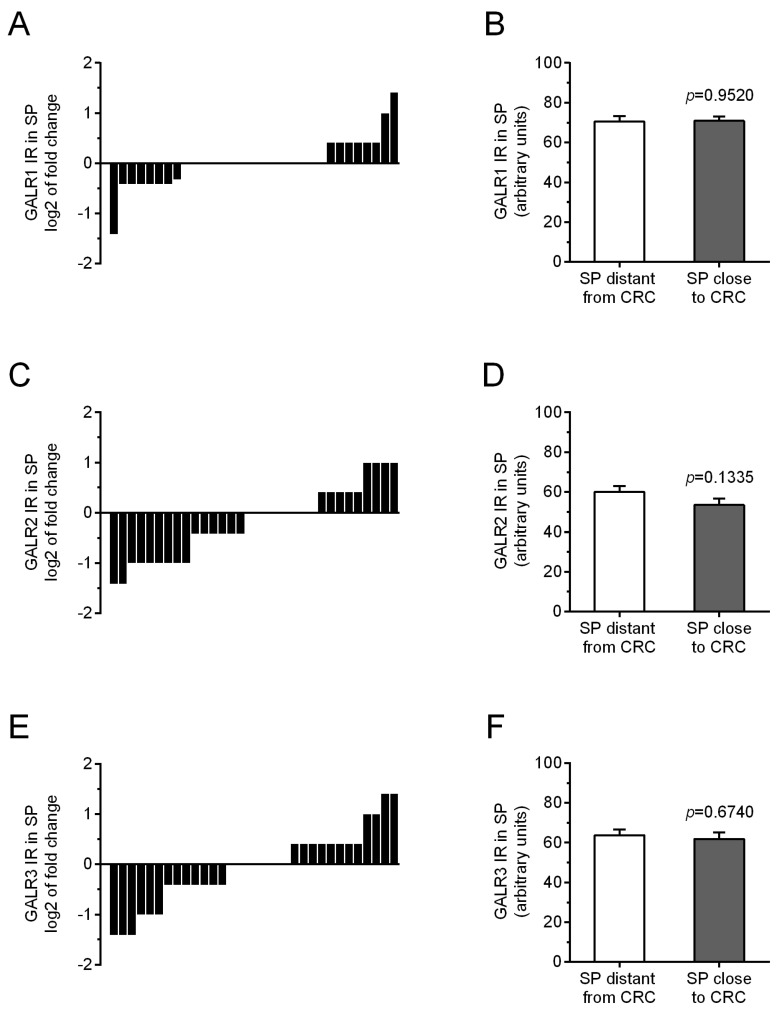
Immunoreactivity (IR) of galanin receptors (GALR1—(**A**), GALR2—(**C**), and GALR3—(**E**)) in submucosal plexuses (SP) distantly and in proximally located to cancer invasion in individual colorectal (CRC) patients. The average immunoreactivities of GALRs (GALR1—(**B**), GALR2—(**D**), and GALR3—(**F**)) in submucosal plexuses (SP) located distantly from CRC tissue were compared with those in plexuses in the vicinity of cancer cells of colorectal (CRC) patients (n = 31).

The immunoreactivity of GALRs in submucosal plexuses in the vicinity of CRC was unchanged as compared with their immunoreactivity in plexuses of unchanged tissue in the same CRC patients (Figure 6). The average immunoreactivity of GALR1 was significantly unchanged (*p* = 0.9520), similarly as immunoreactivity of GALR2 (*p* = 0.1335), and GALR3 (*p* = 0.6740).

### 2.5. Fluorescent Immunolocalization of GALRs in Submucosal Plexuses Located Distantly and Closely to CRC Tissue

**Figure 7:**
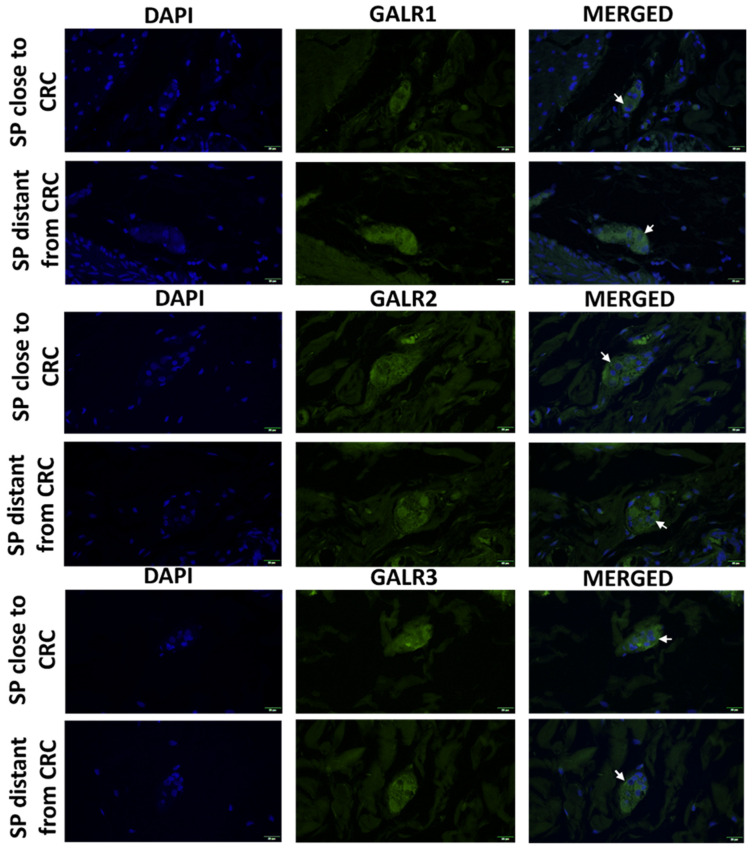
Immunolocalization of galanin receptors (GALR1, GALR2, and GALR3) in submucosal plexuses (SP) close and distant to tumour tissue of colorectal cancer patients (n = 5). Neurons expressing respective GALRs are marked by arrows. Total magnification: 400× *g*.

The immunoexpression of GALR 1, 2 and 3 was confined to the cell membrane and cytoplasm of submucosal plexuses cells and stromal cells (Figure 7).

### 2.6. Immunoexpression of GALR1 in Submucosal Plexusescorrelates with the Prognosis of CRC Patients

**Figure 8:**
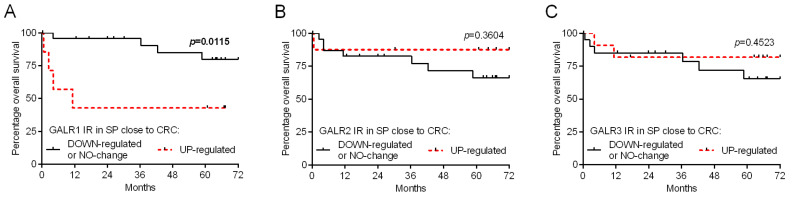
Kaplan–Meier diagrams that show the relative immunoexpression of galanin receptors (GALR1—(**A**), GALR2—(**B**), and GALR3—(**C**)) in submucosal plexuses (SP) regarding the overall survival of colorectal cancer (CRC) patients (n = 32).

We divided CRC patients based on the relative immunoreactivity of GALR specimens into two groups regarded as ‘down-regulated or no change’ (relative GALR immunoreactivity ≤ 1) and ‘up-regulated’ (relative GALR immunoreactivity > 1) to obtain survival curves according to the Kaplan–Meier method. We found that GALR1, but not GALR2 and GALR3, relative immunoreactivity (comparing submucosal plexuses close to CRC cells vs. plexuses distant from cancer invasion) correlate with the overall survival of CRC patients (*p* = 0.0115 and HR = 4.97; Figure 8A; Table 3) and clinical-pathological data of CRC patients. Moreover, the elevated immunoexpression of GALR1 in submucosal plexuses close to cancer correlated with the status of the primary tumour (T), presence of distant metastasis and TNM stage (Table 4).

## 3. Discussion and Conclusions

Galanin and its specific receptors form the galaninergic system, which is expressed in normal tissue, as well as in cancer cells and it seems to be an important molecular factor involved in cancerogenesis, metastasis, and invasion [13]. Our group was the first to report a higher level of GAL in the blood serum of 68 CRC patients, than in healthy volunteers [16]. Moreover, we also found that the highest level of GAL was observed in homogenates of both CRC tumour tissue and samples of mucosa with submucosa located distantly from the tumour. Although the myenteric plexuses in the vicinity of the CRC tissue were much smaller than far from the tumour, the concentration of GAL protein in homogenates of muscularis externa (containing myenteric plexuses) in the vicinity of the CRC tissue was higher than in the samples distant to cancer cells [16]. These studies seemed to be consistent with data describing that neurons of atrophic myenteric plexuses located close to cancer cells express the elevated presence of GAL compared to those located distantly in 15 CRC patients [13], suggesting that smooth muscle cells of muscularis externa may be the target of GAL in the large intestine wall. However, not only cells of muscularis externa but also cells present in the mucosa and submucosa of the large intestine (epithelial cells, stromal/immune cells, immune cells, or neurons) may produce GAL [13]. We decided to analyse the immunoexpression of three types of GAL receptors in the plexuses of large intestine submucosa and muscularis externa of CRC patients in two locations: close and distant from the tumour invasion tissue.

To the best of our knowledge, our present study is the first to demonstrate a similar expression of GALRs proteins in myenteric and submucosal plexuses located in proximity to the tumour tissue compared with myenteric plexuses located distantly from neoplastic tissue in CRC patients, and the immunoreactivity of GALRs in myenteric plexuses was not associated with overall survival and TNM stages. Ciurea et al. (2017) reported, that with an increase in the cancer clinical stage, the size of both enteric plexuses (myenteric and submucosal plexuses) decreases. Atrophy of myenteric plexuses in CRC patients is evident [23]. Atrophy of plexuses in gastric cancer is not caused by apoptosis or necrosis [23]. In our study, we showed similar and slightly decreases GALRs immunoexpression in myenteric plexuses close to the tumour, which was not correlated with tumour grading. High GAL expression in muscularis externa located close to the tumour, as well as in cancer cells [16] and the different situation in our present data with distribution of GALRs in myenteric plexuses, which are not down- or up-regulated by GAL, may indicate that the atrophic plexuses did not respond to the locally increased synthesis of GAL. When myenteric plexuses undergo morphological and functional alterations characteristic for atrophy, GALRs maintain or only slightly decrease their status and do not respond to the disturbance of homeostasis during the invasion of cancer.

In the present study, we demonstrated for the first time the similar expression of GALRs in submucosal plexuses in the vicinity of neoplastic tissue as well as far from the cancer cells—in morphologically unchanged tissue, although well-described atrophy of submucosal plexuses in CRC [15,23,24]. The expression of galanin receptors in plexuses of submucosa was high and, interestingly, higher GALR1 expression in submucosal plexuses was associated with higher tumour size, advanced local invasion, distant metastasis and shorter overall survival. An increasing level of GALR1 in submucosal plexuses in patients characterised by CRC progression and worse prognosis suggests that this receptor acts as an element in pro-proliferative pathways that include neural and neuro-paracrine signalling and may accelerate the invasion and metastasis of cancer cell. Interestingly, in our previous study we have shown previously GAL expression in submucosal plexuses was high [16]. It has been suggested that GAL can act on the tissues by GALR1 in an autocrine manner [25]. Neurons of submucosa plexuses transduce signals to epithelial tissue through secretomotory axons [26]. High GALR1 expression in submucosal plexuses located close to the tumour in patients with poor prognosis may suggest that this GAL receptor is involved in the modulation of cancer cell proliferation. GAL acting through GALR1 exerts both anti- and proliferative effects in different cancer types. The galaninergic system inhibits proliferation in head and neck squamous cell carcinoma (HNSCC), glioma, and gastric cancer cells [27,28,29]. In CRC patients, a high GAL mRNA expression was related to tumour recurrence, and CRC patients in stage II had a poorer prognosis than those showing a low expression of the peptide. [30]. However, Taalat et al., 2022 showed in a recent study on Northern African individuals that GAL protein downregulation is correlated with advanced CRC stages, which is connected with cell cycle regulation, autophagy, and immune system response [31]. In other cancers, epigenetic hypermethylation of *GAL* and *GALR1* genes correlated with bigger tumour size of HNSCC patients [32,33] and activation of GALR1 in SH-SY5Y neuroblastoma cell line induced cell cycle arrest [34]. The siRNA-mediated silencing of the GAL gene reduced both proliferative and invasive potential in CRC cell lines [35]. GALR1 triggers PI3K/Akt-dependent pathway modulates cell proliferation [36]. Silencing of GALR1 in the HCT116 CRC cell line activates apoptosis, and, for more, the GALR1 mRNA level is increased in patients with better prognosis in the early stages of CRC [35]. However, it has to be noted that the up-regulated mRNA levels do not always correspond to the increased protein expression. Enteric neurons also interact with the extensive intrinsic immune system of the gastrointestinal tract [26]. GALRs show a high expression in human glioma and pituitary adenoma tumour-infiltrating immune cells [12], and GAL was found to regulate the expression of chemokines (CCL2, CCL3, CCL5) [13,37]. The neurons of submucosa plexuses expressing GALR1 may activate the release of factors related to the activation of cancer cell proliferation and metastasis by tumour-infiltrating immune cells. Our recent study suggested a high concentration of GALRs in immune/stromal cells of mucosa that may be associated with the modulation by GAL tumour-infiltrating macrophages activation [37].

The higher expression of GALR1 in plexuses near the cancer tissue in CRC patients with poorer prognosis and shorter survival may imply that this receptor in submucosal plexuses can be an important predictive factor for CRC progression. In our previous study, we showed that the GALR3 immunoexpression in CRC cells may be regarded as a prognostic factor. Expressions of GALR1 and GALR3 in cancer cells were higher in comparison to unchanged tissue, however, only GALR3 expression was associated with prognosis and overall survival [1]. Our present study adds important clues to the previous findings by analysing the submucosal compartment of the colon wall of CRC patients. Cancer cells are one of the main sources of GAL and contain large amounts of GALR1 and GALR3 [1,16]. GALR1 and GAL3 share similar signalling pathways, but in our previous study, there was a lack of association between GALR1 expression and CRC prognosis [1]. Similarly, in the submucosal plexuses, only higher expression of GALR1 correlated with higher TNM stages and shorter survival. It may suggest that signalling of the submucosal plexuses neurons rich in GALR1 can activate cancer cell proliferation, invasion and metastasis. The obtained results suggest a crucial role of GALR1 in submucosal plexus function during CRC progression and may imply its role as a predictive factor in this cancer. In future studies, the GALRs expression in the colon wall of CRC patients should include the distribution of those receptors in immune-infiltrating cells in intra- and peritumoural stroma. We suggest that the evaluation of other ligands of GALRs (e.g., spexin) is important to explain the role of the galaninergic system in large intestine cancerogenesis.

## 4. Materials and Methods

### 4.1. Patients

Extracted colorectal tissues were separated postoperatively from the colon wall of CRC patients and paired tissues were obtained from CRC tissue and unchanged large intestine wall of the same patient with histologically confirmed CRC. The paired tissues with visible muscularis externa (contains myenteric plexuses), or submucosa (contains plexuses of submucosa) were selected from 55 CRC patients operated on at the Warmia and Mazury Oncological Centre (Olsztyn, Poland) between 2012 and 2016. A total of 31 CRC patients was included to analysed myenteric plexuses and 32 CRC patients were chosen to evaluate plexuses in the submucosa. None of the CRC patients suffered from inflammatory bowel disease (IBD) or other gastrointestinal diseases, and no patient confirmed a family history of malignancy. None of the patients had suffered from a second neoplastic disease. Patients that had undergone neoadjuvant radiotherapy or chemotherapy were forbidden from the study. The patient’s clinical characteristics and overall survival (OS) data were collected during the study. The tumour stage was indicated according to the TNM system. The study was authorized by the Bioethical Commission of the University of Warmia and Mazury (Olsztyn, Poland, approval no. 43/2011), and all patients gave their written consent to participate in the study.

### 4.2. Collection of Tumour and Colon Wall Samples

Immediately after resection of the part of the large intestine, full-thickness intestinal wall samples (size ~5–6 mm) were collected and separated into two parts: one sample was obtained from the colon wall directly from the tumour and the second control sample was obtained from the proximal part of the intestine at a distance of min. ≥ 5 cm from the tumour. Immediately after dissection, the sections of the colon wall were washed in phosphate-buffered saline (PBS) and fixed in 4% buffered paraformaldehyde for 48 h at room temperature (RT) and frozen in liquid nitrogen for immunofluorescence analysis. In the next step, tissue samples were fixed in paraformaldehyde dehydrated in increasing ethanol series (60%, 90%, 96%, 99.9%), cleared with xylene, embedded in paraffin and cut into 5 µm-thick tissue slices.

### 4.3. Immunohistochemistry and Immunofluorescence

Immunohistochemical analysis was performed as described previously by Kieżun et al. (2022) with modifications. The sections were subjected to an antigen retrieval procedure by microwaving for 12 min in Retrieval Solution Buffer, pH 6.0 (Leica Microsystems, Wetzlar, Germany), and then incubation with 3% H_2_O_2_ in methanol for 10 min for blocked endogenous peroxidase activity. Next, the unspecific binding sites were blocked with 2.5% normal horse serum (Vector Laboratories, Burlingame, CA, USA) for 30 min. The sections were incubated overnight at 4 °C with rabbit polyclonal anti-human antibodies against GALR1 (Cat. No. GTX108207, GeneTex, Irvine, CA, USA), GALR2 (Cat. No. GTX100382, GeneTex, Irvine, CA, USA) and GALR3 (Cat. No. GTX10816, GeneTex, Irvine, CA, USA), all diluted 1:400 in PBS. On the next day, sections were incubated with secondary antibodies (ImmPRESS Universal reagent Anti-Mouse/Rabbit Ig, Vector Laboratories, Burlingame, CA, USA) for 30 min. Schrődl et al. (2015) checked that there were no cross-reactions between the GALR1 and GALR3 antibodies [38] that were also used in our study. The specificity of immunohistochemical staining was checked by omitting the primary antibody and by replacing it with the rabbit serum. To visualize the immunoreaction, the sections were visualized in DAB (Dako, Carpinteria, CA, USA), then counterstained with hematoxylin (Sigma-Aldrich, St. Louis, MO, USA), dehydrated in ethanol series, rinsed in xylene and mounted in DPX (Sigma-Aldrich, St. Louis, MO, USA). The immunostained sections were photographed using an Olympus BX-53 microscope and XC-50 camera (Olympus Corp., Tokyo, Japan).

The immunoreactivity of GALRs was assessed by two different scientists, who were blinded to the patient clinical data, in the membrane and cytoplasm of the cells of enteric plexuses close to CRC cells and enteric plexuses of unchanged tissue, using the scale based on the reaction intensity (0, no reaction; 10, ≤10%; 30, 11–30%; 60, 31–60%; 80, 61–80%; and 100, >80%) as described previously [39]. In the cases of different assessments, the third scientist checked the sections.

The immunofluorescent staining was performed as described by Kisielewska et al. (2020) [40]. Briefly, the unchanged tissue and tumour tissue (n = 5) were washed, fixed and cryo-protected for 1–2 days in graded solutions (19% and 30%) of sucrose (Sigma-Aldrich, St. Louis, MO, USA) at 4 °C. The tissues were frozen, cut into 5 μm thick cryostat coronal sections, and stored at −80 °C. The sections were air-dried, washed and incubated for 1 h with blocking buffer (0.1 M PBS, 10% normal donkey serum, 0.01% bovine serum albumin, 1% Tween, 0.05% thimerosal and 0.01% NaN3). Then, the sections were incubated overnight with rabbit polyclonal antibodies diluted at the same concentrations as in the case of the immunohistochemistry method and then for 1 h with the Alexa Fluor 555 donkey anti-rabbit antibodies (1:1000, A-31572, Molecular Probes, Eugene, OR, USA). To visualize the nuclei, the sections were stained with DAPI mounted in Fluoroshield (Merck, Kenilworth, NJ, USA). The sections were photographed using an Olympus BX-53 microscope with an Olympus DP23 camera and analysed by CellSense v3.2 software (Olympus Corp., Tokyo, Japan). The specificity of immunofluorescent staining was checked by omitting the primary antibody.

### 4.4. Statistical Analyses

All statistical analyses regarding the immunoexpression of GALR proteins by immunohistochemistry were performed using Statistica software version 13.3 (Tibco Software Inc., Palo Alto, CA, USA) and Prism 6.07 (GraphPad Software, San Diego, CA, USA). The results were expressed as the mean ± standard error of the mean (SEM). *p* < 0.05 was considered to indicate a statistically significant difference. The differences between the immunoreactivity of GALRs in myenteric plexuses and plexuses of submucosa distantly located from the CRC tissue compared with their expression in these receptors in the vicinity of neoplasm invasion of the same, representative colorectal (CRC) patients were detected by a Wilcoxon matched-pairs test. The Fisher’s exact test and Chi^2^ test were used to assess associations between clinico-pathological data and the immunoexpression of GALRs in studied tissues of the same CRC patients. Survival curves were plotted according to the Kaplan–Meier method. The hazard ratio (HR) and the significance of difference in overall survival (OS) between groups of patients were calculated using log-rank test (Prism, GraphPad Software).

## Figures and Tables

**Table 1:** Analysis of overall survival of CRC patients in relation to GALRs immunoexpression in myenteric plexuses. HR-hazard ratio; CI-confidence interval; MP-myenteric plexus.

Covariates	HR	95% CI	*p*-Value
GALR1 in MP close to CRC			
UP regulated	2.03	0.35–18.30	0.3612
DOWN-regulated or NO-change	1.00		
GALR2 in MP close to CRC			
UP regulated	3.39	0.96–48.62	0.0575
DOWN-regulated or NO-change	1.00		
GALR3 in MP close to CRC			
UP regulated	0.42	0.11–2.40	0.4019
DOWN-regulated or NO-change	1.00		

**Table 2:** Associations between demographic and clinico-pathological features of colorectal cancer patients (n = 31) and relative expression levels of galanin receptors in myenteric plexuses determined by immunohistochemistry.

Qualitative Parameters	Number of Cases	GALR1-IR in Myenteric Plexuses					GALR2-IR in Myenteric Plexuses					GALR3-IR in Myenteric Plexuses				
		Down-Regulated or No Change		Up-Regulated		*p*-Value	Down-Regulated or No Change		Up-Regulate		*p*-Value	Down-Regulated or No Change		Up-Regulated		*p*-Value
**Total**	31	28	90.3%	3	9.7%		26	83.9%	5	16.1%		26	83.9%	5	16.1%	
male	19	17	89.5%	2	10.5%	1.0000	16	84.2%	3	15.8%	1.0000	16	84.2%	3	15.8%	1.0000
female	12	11	91.7%	1	8.3%		10	83.3%	2	16.7%		10	83.3%	2	16.7%	
**Age**																
≤median (66 years old)	16	14	87.5%	2	12.5%	1.0000	13	81.3%	3	18.8%	1.0000	13	81.3%	3	18.8%	1.0000
>median (66 years old)	15	14	93.3%	1	6.7%		13	86.7%	2	13.3%		13	86.7%	2	13.3%	
**Localization**																
cecum. right colon	8	7	87.5%	1	12.5%	0.0980	7	87.5%	1	12.5%	0.3081	7	87.5%	1	12.5%	0.3081
transverse. left colon. sigmoid	16	16	100.0%	0	0.0%		12	75.0%	4	25.0%		12	75.0%	4	25.0%	
rectum	7	5	71.4%	2	28.6%		7	100.0%	0	0.0%		7	100.0%	0	0.0%	
**T status**																
T2+T3	24	21	87.5%	3	12.5%	1.0000	20	83.3%	4	16.7%	1.0000	20	83.3%	4	16.7%	1.0000
T4	7	7	100.0%	0	0.0%		6	85.7%	1	14.3%		6	85.7%	1	14.3%	
**N status**																
N0	13	11	84.6%	2	15.4%	0.5575	11	84.6%	2	15.4%	1.0000	11	84.6%	2	15.4%	1.0000
N1+N2	18	17	94.4%	1	5.6%		15	83.3%	3	16.7%		15	83.3%	3	16.7%	
**Distant metastases**																
M0	25	22	88.0%	3	12.0%	1.0000	22	88.0%	3	12.0%	0.2406	22	88.0%	3	12.0%	0.2406
M1	6	6	100.0%	0	0.0%		4	66.7%	2	33.3%		4	66.7%	2	33.3%	
**TNM stage**																
I+II	11		0.0%	2	18.2%	0.5831	11	100.0%	2	18.2%	0.9675	9	52.9%	8	47.1%	0.3711
III	12		0.0%	1	8.3%		11	91.7%	2	16.7%		8	80.0%	2	20.0%	
IV	5		0.0%	0	0.0%		4	80.0%	1	20.0%		3	60.0%	2	40.0%	

IR—immunoreactivity.

**Table 3:** Analysis of overall survival of CRC patients in relation to GALRs immunoexpression in submucosal plexuses. HR-hazard ratio; CI-confidence interval; SP-submucosal plexus.

Covariates	HR	95% CI	*p*-Value
**GALR1 in SP close to CRC**			
UP regulated	**4.97**	1.72–70.40	**0.0115**
DOWN-regulated or NO-change	1.00		
**GALR2 in SP close to CRC**			
UP regulated	0.39	0.10–2.30	0.3604
DOWN-regulated or NO-change	1.00		
**GALR3 in SP close to CRC**			
UP regulated	0.55	0.14–2.41	0.4523
DOWN-regulated or NO-change	1.00		

**Table 4:** Association between demographic and clinico-pathological features of colorectal (CRC) patients (n = 32) and relative expression levels of galanin receptors in submucosal plexuses determined by immunohistochemistry.

Qualitative Parameters	Number of Cases	GALR1-IR in Submucosal Plexuses					GALR2-IR in Submucosal Plexuses					GALR3-IR in Submucosal Plexuses				
		Down-Regulated or No Change		Up-Regulated		*p*-Value	Down-Regulated or No Change		Up-Regulated		*p*-Value	Down-Regulated or No Change		Up-Regulated		*p*-Value
**Total**	32	24	75.0%	8	25.0%		23	71.9%	9	28.1%		20	62.5%	12	37.5%	
male	19	14	73.7%	5	26.3%	1.0000	14	73.7%	5	26.3%	1.0000	15	78.9%	4	21.1%	0.0300
female	13	10	76.9%	3	23.1%		9	69.2%	4	30.8%		5	38.5%	8	61.5%	
**Age**																
≤median (66 years old)	17	15	88.2%	2	11.8%	0.1058	13	76.5%	4	23.5%	0.6989	12	70.6%	5	29.4%	0.4670
>median (66 years old)	15	9	60.0%	6	40.0%		10	66.7%	5	33.3%		8	53.3%	7	46.7%	
**Localization**																
cecum. right colon	12	11	91.7%	1	8.3%	0.1119	9	75.0%	3	25.0%	0.9325	9	75.0%	3	25.0%	0.4111
transverse. left colon. sigmoid	14	8	57.1%	6	42.9%		10	71.4%	4	28.6%		7	50.0%	7	50.0%	
rectum	6	5	83.3%	1	16.7%		4	66.7%	2	33.3%		4	66.7%	2	33.3%	
**T status**																
T2+T3	25	22	88.0%	3	12.0%	**0.0048**	17	68.0%	8	32.0%	0.6401	17	68.0%	8	32.0%	0.3793
T4	7	2	28.6%	5	71.4%		6	85.7%	1	14.3%		3	42.9%	4	57.1%	
**N status**																
N0	17	14	82.4%	3	17.6%	0.4235	12	70.6%	5	29.4%	1.0000	9	52.9%	8	47.1%	0.2907
N1+N2	15	10	66.7%	5	33.3%		11	73.3%	4	26.7%		11	73.3%	4	26.7%	
**Distant metastases**																
M0	26	22	84.6%	4	15.4%	**0.0228**	18	69.2%	8	30.8%	0.6482	16	61.5%	10	38.5%	1.0000
M1	6	2	33.3%	4	66.7%		5	83.3%	1	16.7%		4	66.7%	2	33.3%	
**TNM stage**																
I+II	17	14	82.4%	3	17.6%	**0.0076**	12	70.6%	5	29.4%	0.9073	9	52.9%	8	47.1%	0.3711
III	10	9	90.0%	1	10.0%		7	70.0%	3	30.0%		8	80.0%	2	20.0%	
IV	5	1	20.0%	4	80.0%		4	80.0%	1	20.0%		3	60.0%	2	40.0%	

IR—immunoreactivity.

## Data Availability

The data presented in this study are available on request from the corresponding author.
